# TULA Proteins in Men, Mice, Hens, and Lice: Welcome to the Family

**DOI:** 10.3390/ijms24119126

**Published:** 2023-05-23

**Authors:** Alexander Y. Tsygankov

**Affiliations:** Sol Sherry Thrombosis Research Center, Lewis Katz School of Medicine, Temple University, Philadelphia, PA 19140, USA; tsygan@temple.edu

**Keywords:** TULA, TULA-1, TULA-2, Sts-1, Sts-2, UBASH3A, UBASH3B, Syk, ZAP-70

## Abstract

The two members of the UBASH3/STS/TULA protein family have been shown to critically regulate key biological functions, including immunity and hemostasis, in mammalian biological systems. Negative regulation of signaling through immune receptor tyrosine-based activation motif (ITAM)- and hemITAM-bearing receptors mediated by Syk-family protein tyrosine kinases appears to be a major molecular mechanism of the down-regulatory effect of TULA-family proteins, which possess protein tyrosine phosphatase (PTP) activity. However, these proteins are likely to carry out some PTP-independent functions as well. Whereas the effects of TULA-family proteins overlap, their characteristics and their individual contributions to cellular regulation also demonstrate clearly distinct features. Protein structure, enzymatic activity, molecular mechanisms of regulation, and biological functions of TULA-family proteins are discussed in this review. In particular, the usefulness of the comparative analysis of TULA proteins in various metazoan taxa, for identifying potential roles of TULA-family proteins outside of their functions already established in mammalian systems, is examined.

## 1. Novel Family of Atypical Protein Tyrosine Phosphatases

The UBASH3 gene/protein family was defined through the independent effort of several research groups approximately 20 years ago. The first report on a UBASH3 family member published in 2001 was focused on characterization of human UBASH3A, a novel gene, whose possible involvement in the Down syndrome-related autosomal recessive deafness was ruled out in this study [1]. The sequence and tissue expression of UBASH3A transcript was determined in these experiments. A study describing identification, cloning, and targeted deletion of p70, a novel mouse protein that appeared to be related to UBASH3A, was then published in 2002 [2]. This report was followed by a study from the same group characterizing what was previously termed UBASH3A in great detail [3]. Two other studies, focusing on the characterization of UBASH3A protein, were reported soon [4,5]. Thus, the new protein family, which contained two members, had been defined (reviewed in [6,7]).

The genes and proteins of this family are annotated in databases as UBASH3A and UBASH3B due to the presence of ubiquitin-associated (UBA) and Src-homology 3 (SH3) domains in their structure. Several synonyms have been introduced to denote them in the original reports, and these synonyms are frequently used. UBASH3A has been termed STS-2 for Suppressor of T-cell Signaling, TULA (or TULA-1) for T-cell Ubiquitin Ligand, and CLIP4 for Cbl-Interacting Protein 4. UBASH3B has been termed STS-1, TULA-2, and p70. In this publication, the terms TULA-1 and TULA-2 are always used, regardless of the term used in the original papers, solely for the sake of consistency with our other publications and throughout this review.

## 2. TULA Protein Domains and Their Functions

**Figure 1:**
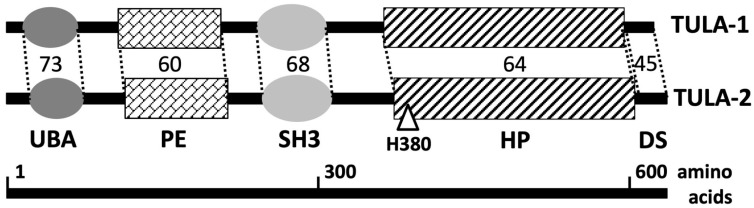
Structure of TULA-family proteins. Major functional domains of TULA proteins are shown, including ubiquitin-associated domain (UBA), Src-homology domain 3 (SH3), 2H phosphoesterase (PE) domain, and histidine phosphatase (HP) domain—the dimerization sequence (DS). The degree of protein sequence homology within the major regions is shown as a percentage of the identical plus similar (‘positive’) amino acid residues. The key histidine residue of the HP domain is indicated (H380 in mouse TULA-2); length and amino acid numbering corresponds to the protein sequence of mouse TULA-family proteins.

The structure of TULA-family proteins exhibits a unique combination of functional domains, which includes UBA and SH3 interactive domains and the histidine phosphatase domain (Figure 1). The UBA and SH3 domains are generally well characterized, and it has been shown that they contribute to the functions of TULA-family proteins. The SH3 domain mediates binding of TULA-family members to various SH3-binding proteins [4,5,8]. The UBA domain binds to ubiquitin and various ubiquitylated proteins [4,5,9,10,11,12], including TULA-family proteins themselves when they become ubiquitylated [10]. TULA proteins bind to other proteins as well [13,14,15,16,17,18], but the specific domains mediating this binding have not been identified.

The C-terminal region of TULA proteins was initially recognized as an area of homology (i) between the TULA orthologues from human, *C. elegans* and Drosophila [1] and (ii) to mammalian phosphoglycerate mutase [2]. Subsequently, this region has been shown to be a histidine phosphatase (HP) domain [19], which plays a key functional role in both TULA-family proteins [14,19,20,21]. The histidine phosphatase superfamily is a large and diverse group of proteins, which is unified by the presence of a key catalytic histidine residue in the active site of these enzymes. This superfamily includes both various phosphatases and non-phosphatase enzymes, such as phosphoglycerate mutases [22].

In the TULA family, the HP domain exhibits the ability to hydrolyze multiple low-molecular weight phosphatase substrates, phosphotyrosine (pY)-containing peptides, and pY-containing proteins [14,19,20,23,24]. The latter appear to be the major physiological substrates of this phosphatase family, and thus can be considered an atypical protein tyrosine phosphatase (PTP) family, since the structure of TULA-family PTPs is different from the traditional PTPs, where cysteine plays the role of a key catalytic residue [25,26,27].

The C-terminal-most region of TULA proteins mediates their dimerization, which has been established using size-exclusion chromatography [28], crystal structure analysis [19], gel-electrophoresis with and without prior chemical cross-linking [5], and co-immunoprecipitation of differentially tagged subunits [14]. Despite these findings, the functional significance of TULA oligomerization remains to be understood.

The latest addition to this family’s set of functional domains is a 2H phosphoesterase domain, termed this way due to the presence of two histidine residues that are essential for its phosphodiesterase activity [29]. The 2H domain of TULA-2 exhibits enzymatic activity toward 2′,3′ cyclic NADP and a 5-mer RNA oligonucleotide containing a 2′,3′ cyclic phosphate group [30]. Although natural substrates of this enzymatic domain remain unidentified, it appears that phosphodiesterase activity participates in the biological effect of TULA-2 [30].

Functional roles of TULA domains have been analyzed in multiple and diverse experimental systems. Briefly, the HP domain and its role have been studied very thoroughly. It has been shown that HP is essential for the functions of the TULA family. Mutations inactivating its PTP activity impair the ability of TULA-2 to suppress signaling and cell activation mediated by the Syk-family of protein tyrosine kinases (PTK), which in T cells, where the original studies have been conducted, is represented by ZAP-70 [14,19,20,21,31]. The key role of PTP activity in these processes appears to be mediated by dephosphorylation of Syk-family PTKs, which are essential for signaling in the systems where these experiments have been conducted. The SH3 and UBA domains also contribute to the effects of TULA proteins [14,15,19], although the molecular mechanism of this contribution is less clear; it may be linked to substrate binding or sub-cellular localization, or be mediated by PTP-independent functions of TULA proteins (reviewed in [32]). More details related to these issues can be found below in the sections focused on the substrates and biological functions of TULA proteins. Finally, the ability of TULA-2 to suppress T-cell receptor (TCR)-mediated T-cell activation is inhibited by inactivating mutations of the 2H domain’s key histidines, indicating an essential role of this domain as well, although the molecular basis of this effect remains to be demonstrated [30].

The two TULA-family members demonstrate a substantial degree of the overall amino acid sequence similarity (~45% identical residues), which is only slightly lower than that in such two-member families as the Syk/ZAP-70 PTKs or the SHP-1/SHP-2 PTPs (~55% and ~60% identical residues, respectively). Expectedly, inside the functional domains, sequence similarity between TULA-1 and TULA-2 is even greater (see Figure 1).

## 3. TULA-Family Tissue and Cell Type Expression

Despite the structural similarities, differences between the two family members are also apparent. The initial studies demonstrated that TULA-2 is ubiquitously expressed in various cells and tissues [2,3]. It was later shown that the expression of this family member is especially high in platelets [16]. In contrast, cell/tissue expression of TULA-1 was immediately demonstrated to be specific; it is highly expressed in lymphoid tissues, including thymus and spleen, but not in multiple non-lymphoid tissues analyzed [3,4]. Furthermore, while the levels of TULA-2 in T and B cells were found to be similar, the level of TULA-1 in T cells substantially exceeded that in B cells [3]. The presence of TULA-1 mRNA was detected in some non-lymphoid tissues using PCR (of them, the lung exhibited the highest level), but this relatively low transcript level could be due to lymphocyte infiltrations [1,4].

Despite the initial negative results, expression of TULA-1 was later demonstrated in myeloid cells which mediate innate immune responses: monocytes/macrophages, neutrophils, and dendritic cells [33]. This issue is complicated by the apparent dependence of TULA-1 expression on the developmental/activation state of these cells; a difference in TULA-1 and TULA-2 expression between adherent and non-adherent populations of ex vivo differentiating monocytes/macrophages has been observed [33]. Notably, these developmental changes may be opposite for the two family members; bone marrow-derived dendritic cells have been shown to increase the level of TULA-2 while losing TULA-1 expression [33]. In agreement with these changes in TULA-family expression, the expression of TULA-2 is up-regulated over the course of differentiation of osteoclasts, which are macrophage-related cells [21].

## 4. PTP Activities of TULA-Family Proteins

Another noted difference between the two family members is related to their specific PTP activity. A high degree of sequence similarity between TULA-family members, especially inside their HP domain (see Figure 1), consistent with the conserved nature of the key amino acid residues of the PTP active site [20,23], strongly supports the idea that the effects of both family members depend on their PTP activity. However, mouse TULA-2 is consistently more enzymatically active than mouse TULA-1 toward a wide variety of substrates (low-molecular weight synthetic molecules, pY-peptides, and pY-proteins) and under widely varying reaction conditions [11,19,24]. Although the difference between human TULA-2 and TULA-1 PTP activities is less profound than that for the mouse pair, human TULA-2 remains substantially more active than human TULA-1 [34].

In agreement with these findings, a library screening using peptides containing a central pY residue surrounded by random flanking sequences easily yielded multiple pY-peptide substrates of TULA-2 (see below for details), although no pY-peptide was identified to be dephosphorylated by TULA-1 [35]. Furthermore, a study in a co-transfection model system demonstrated that both the PTP-inactivated mutant TULA-2 and wild-type (WT) TULA-1 similarly interfere with a dephosphorylation effect of WT TULA-2 on Syk, acting in a dominant-negative manner. This is consistent with the dramatic difference in PTP activities between WT TULA-2, on the one hand, and PTP-inactivated TULA-2 and WT TULA-1, on the other hand [14]. Together, these results indicate that the intrinsic PTP activity of TULA-2 is much higher than that of TULA-1.

## 5. Specific Functions of Individual Family Members in the Immune System

Despite a significant disparity in PTP activity between the two family members, their key cellular functions overlap. Indeed, a very strong up-regulatory effect of the TULA-family double knockout (dKO) on TCR-mediated signaling, proliferation, and cytokine secretion in T-cell culture, as well as an exacerbation of severity of experimental autoimmune encephalomyelitis in vivo, was demonstrated in the seminal study characterizing dKO mice [3]; a strong synergism of the two single KOs (sKOs) had been shown, since the effect of either sKO on proliferation was very low as compared to that of dKO [3,19]. It was subsequently demonstrated that, despite a significant difference in specific PTP activities of TULA-family members, both TULA-1 and TULA-2 were capable of dephosphorylating ZAP-70, a key PTK of T-cell signaling, although TULA-2 dephosphorylated it to a higher degree, as expected from the observed difference in PTP activities between the two family members [24].

It should be noted that, although the effects of TULA-1 and TULA-2 deficiency in dKO on various immune responses in vivo and in vitro are clearly synergistic [3,19,36], the individual sKOs also exhibit substantial and specific effects on immune responses [36]. Thus, each sKO exacerbated inflammation in a mouse model of colitis, albeit affecting different parameters. Likewise, each sKO exerted a specific effect on cytokine production in vivo and/or in T cells activated through TCR in vitro. For example, dKO and TULA-2 sKO, but not TULA-1 sKO, facilitated secretion of IL-2 and TNF-α in a culture of T cells from colitis-induced mice, while TULA-1 sKO and dKO, but not TULA-2 sKO, facilitated production of IP-10 in these mice in vivo. Furthermore, only TULA-2 sKO facilitated secretion of IFN-γ in the culture of T cells from colitic mice, while TULA-1 sKO was the only KO facilitating in vivo production of IL-6 [36].

Consistent with the results outlined above, not only dKO, but both TULA-1 and TULA-2 sKO mice demonstrated the improved resistance to systemic Candida infection [37]. The host defense against Candida involves not only T cells, but also innate immune responses mediated by neutrophils, macrophages, and epithelial cells (reviewed in [38,39,40]). The contribution of specific types of immune cells to the protective effect of TULA KOs was not addressed in [37], yet it was later shown that either TULA-family sKO enhances production of nitric oxide by IFN-γ-treated bone marrow-derived monocytes [41], suggesting that the lack of an individual family member may be sufficient for up-regulating responses of cells which mediate innate immunity. Furthermore, the finding that in vivo effects of TULA-2 sKO on inflammation in a mouse model of colitis are, to a large extent, independent of T cells [36] also supports the idea that TULA-2 directly affects innate immunity.

It should be noted that TULA-1 also exerts regulatory effects that appear to be mediated by its interactions with the NF-κB activation pathway in T cells [9,42]. This effect is likely to be very important for T-cell responses, but its molecular basis appears to be PTP-independent (see below for more details).

## 6. Effects of TULA-2 in the Cells Lacking TULA-1

Overall, immunity, especially immunity dependent on T-cell responses, is a major biological phenomenon regulated by TULA-family proteins. However, immunity is not the only major biological function influenced by the TULA family. Platelets have been shown to express TULA-2 at a level substantially exceeding that in other blood cells [16], in agreement with the high level of TULA-2 transcriptional up-regulation over the course of human megakaryocyte development in vitro [43]. This finding, together with the finding that Syk, a key PTK in platelet signaling [44,45,46], is targeted by TULA-2 [14], renders this PTP a critical regulator of platelet activation. In contrast, TULA-1 was not detected in platelets using immunoblotting [16], while proteomic studies indicated that the platelet level of TULA-1 is ~1/10 of that of TULA-2 [47,48].

**Figure 2:**
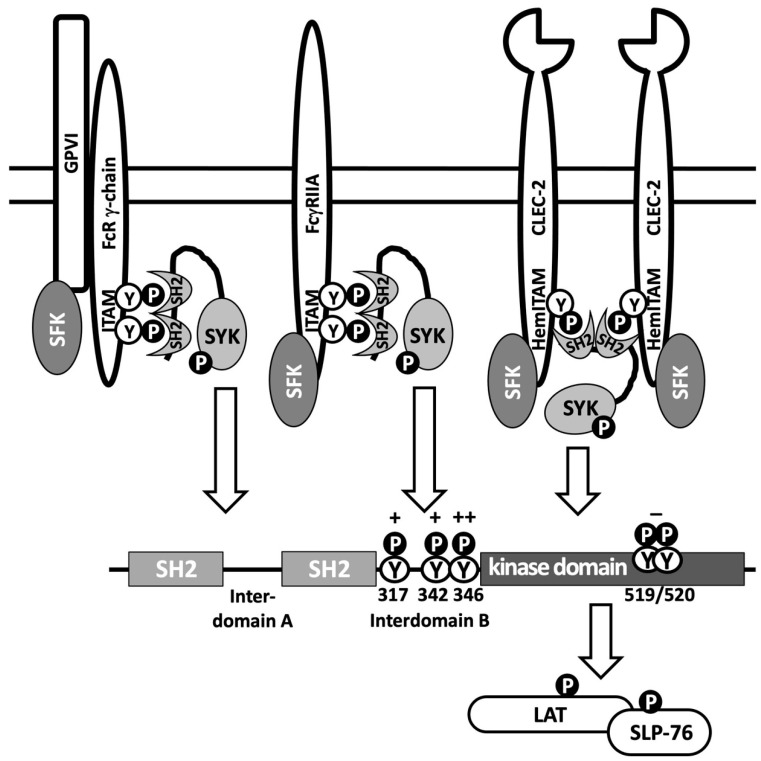
Syk in signaling through ITAM- or hemITAM-bearing receptors. Major upstream events of signaling through ITAM- or hemITAM-bearing receptors are schematically represented using platelet receptors as an example. Receptor-proximal events involve Src-family protein tyrosine kinases (SFK). Syk interacts with phosphotyrosines of ITAMs and hemITAMs through its tandem SH2 domains. This interaction makes Syk tyrosine residues available for phosphorylation by SFK and autophosphorylation by Syk itself. Major regulatory tyrosines of Syk are shown. Under physiological conditions, Y346 is mostly phosphorylated by SFK, while Y317, Y342, and Y519/520 are mostly autophosphorylated. Following activation through a receptor, Syk phosphorylates its main protein substrates, LAT and SLP-76, adaptor proteins which initiate a signal transduction pathway to induce platelet responses. TULA-2 down-regulates Syk-mediated signaling by dephosphorylating several pY-sites of Syk; their sensitivity to TULA-2-dependent dephosphorylation varies from high (++) to appreciable (+) to negligible (−), as indicated. See the text for details.

Consistent with these results, TULA-2 sKO and TULA-family dKO exerted equally strong enhancing effects on platelet signaling and activation, while TULA-1 sKO platelets demonstrated responses indistinguishable from those of WT platelets [49]. The lack of TULA-2 significantly enhanced platelet responses to stimulation through platelet receptors bearing immunoreceptor-based tyrosine activation motif (ITAM), such as the glycoprotein VI (GPVI)/Fc receptor γ-chain (FcR-γ chain) complex [16,35,49] and FcγRIIA [50,51], as well as receptors bearing hemITAMs (hemITAM is a signaling structure resembling one half of an ITAM), such as the C-type lectin-like receptor-2 (CLEC-2) [52], which depend on Syk for transmitting their signals (Figure 2). In contrast, platelet stimulation by thrombin, which depends on receptors coupled to G-proteins, is not regulated by TULA-2 [16,50,51]. The lack of TULA-2 enhanced the entire spectrum of platelet responses in vitro, including aggregation, secretion, integrin activation, and thromboxane production. Importantly, changes in TULA-2 level caused by KO or microRNA-dependent modulation resulted in significant in vivo effects, such as a decrease in tail bleeding time and an increase in thrombotic reactions in model systems [16,50,51]. As a result of these studies, TULA-2 is considered one of the major negative regulators of platelet signaling and activation, as reviewed in detail in [53].

It should be noted that platelets, being a critical element of hemostasis and thrombosis, are also involved in immune responses [54,55,56]. It is therefore possible that the roles of TULA-2 in the regulation of immune and platelet responses overlap to a significant extent at the level of the whole organism. For example, the finding that TULA-2 sKO affects inflammation in a mouse model of colitis largely independent of T cells [36] might be linked to the involvement of platelet responses to this inflammatory process.

The regulatory role of TULA-2 in the bone-resorbing function of osteoclasts, while less extensively studied, clearly resembles that observed in immune cells and platelets. It has been shown that bone volume is decreased in mice lacking TULA-2 due to an increase in both the number of osteoclasts and their activity. This biological effect correlates clearly with a negative effect of TULA-2 on ITAM-mediated signaling required for osteoclast development and function. Consistent with this effect of TULA-2, expression of a PTP-defective form of TULA-2 increased osteoclast activity [21].

## 7. Syk-Family PTKs as a Major Known Biological Target of the TULA Family

In immune cells and platelets, where the role of the TULA family has been examined extensively, the molecular basis of the observed effects is thought to be related to the ITAM-/hemITAM-mediated signaling dependent on ZAP-70 and Syk PTKs, which form a two-member Syk family (reviewed in [32,53,57,58]). Together, these studies point at the dephosphorylation of Syk-family PTKs as a major molecular mechanism in the biological functions of TULA proteins in T cells and platelets. Several lines of evidence indicate that Syk and ZAP-70 are bona fide substrates of TULA-family PTPs. It should be noted, however, that these studies cover TULA-2 to a much higher extent than TULA-1, which is reflective of the higher PTP activity of TULA-2, as well as of the fact that TULA-2 appears to act alone in platelets, so all studies in platelets are focused on TULA-2 exclusively.

**Figure 3:**
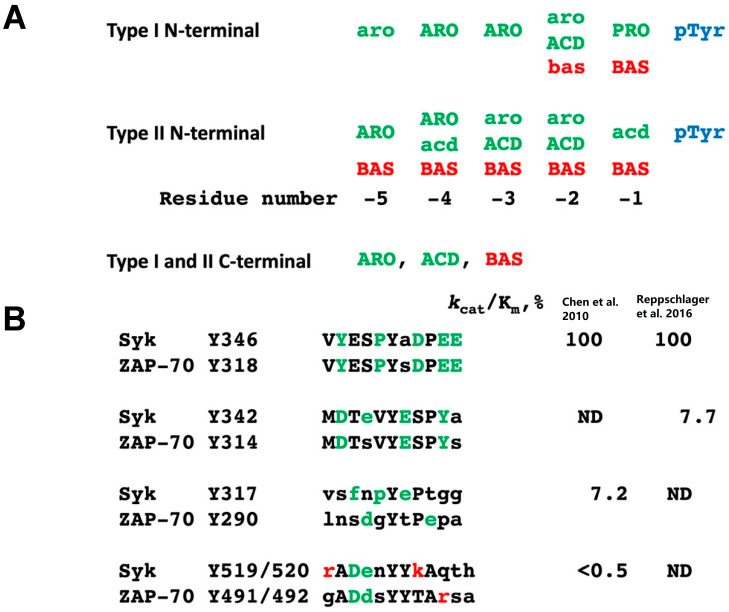
TULA-2 substrate specificity determinants. (**A**) Class I N-terminal motif is defined by a proline residue at position pY-1. It also requires the presence of one or two aromatic residues and the exclusion of basic residues at certain positions. Preference (green) or aversion (red) of various types of amino acid residues at specific positions is shown by the letter case: strong and weaker preference/aversion are indicated as uppercase and lowercase, respectively. Class II N-terminal motif shows a strong preference for aromatic residues at positions pY-5 and pY-4, and acidic residues at pY-3 and pY-2. Positions pY-3 and pY-2 also show some preference for aromatic residues. Also, a very strong aversion to basic residues for the entire length of the analyzed sequence is seen in class II. C-terminal substrate sequences show a strong preference for aromatic and acidic residues, but no clear consensus motif is recognized there. (**B**) Sequences of the major regulatory pY-sites of mouse Syk and ZAP-70 are presented. Homologies between the two PTKs are denoted by uppercase. Positive and negatives substrate specificity determinants are indicated in green and red, respectively. The specificity constants (*k*_cat_/K_m_) for these substrate sites, determined in Refs. [35,49], are indicated. ND = not determined.

(i) TCR-induced phosphorylation of ZAP-70, indicative of the activation of this PTK, is significantly enhanced in T cells which lack TULA proteins. This effect is much stronger in dKO T cells [3,19,24,36]. Notably, the effects of TULA-2 sKO on ZAP-70 phosphorylation and ZAP-70-mediated signaling are substantially more profound than the effects of TULA-1 sKO [24]. However, the PTP-mediated effect of TULA-1 on T-cell signaling is consistent with the following findings: the effect of dKO on ZAP-70 and ZAP-70-mediated signaling is significantly higher than that of TULA-2 sKO [24], TULA-1 has a detectable PTP activity [20,24], and TULA-1 and TULA-2 sKOs exhibit comparable effects on ZAP-70 phosphorylation in T cells from mice with a chemically induced colitis [36];
(ii) Phosphorylation and activation of Syk, as well as Syk-dependent signaling induced by various ITAM-bearing receptors, is significantly enhanced in the cells lacking both TULA proteins or TULA-2 alone [16,21,35] or, in contrast, down-regulated by co-expressing TULA-2 [14] in several experimental systems;
(iii) Screening of libraries containing peptides with a pY residue surrounded by random flanking sequences yielded multiple pY-peptide substrates of TULA-2 and revealed the structural specificity determinants of this PTP [32,35] (Figure 3). Based on these findings, several pY-sites, including Syk pY346 and ZAP-70 pY318, which are highly homologous, have been identified as prime targets of the TULA-2 PTP activity. This conclusion has been validated using individual synthetic pY-peptides bearing various specificity determinants [35] and full-length Syk in vitro [35], as well as activated platelets [35,49], T cells [24,36], and osteoclasts [21];
(iv) WT TULA-2 and its substrate-trapping mutant form bind to Syk and ZAP-70 in a strong and selective fashion in cell lysates from platelets [16,49], T lymphocytes [59], and HEK293T cells [14]. Notably, ZAP-70 and Syk account for most of the pY-containing protein material bound to a substrate-trapping TULA-2 mutant in T cells [59] and platelets [49], respectively. Taken together, these results strongly suggest that the Syk-family PTKs represent major cellular targets of TULA-2.

Although Syk is clearly a key biological substrate of TULA-2, the mode of TULA-2–Syk binding is insufficiently understood. Considering that the SH3 domain of TULA proteins physically interacts with Cbl [4,5], an E3 ubiquitin ligase binding to Syk and mediating its ubiquitylation [60,61,62], and that the UBA domain of TULA proteins binds to ubiquitin [4,5], one may hypothesize that TULA-2 interacts with Syk by binding simultaneously to the Syk-associated Cbl and to the ubiquitin covalently attached to Syk over the course of Cbl-dependent E3 reaction. However, TULA-2 binds to both ubiquitylated and non-ubiquitylated Syk in cell lysates [14,16]. Furthermore, full-length TULA-2 dephosphorylates non-ubiquitylated Syk and various pY-peptides in vitro, and the PTP domain TULA-2, which lacks both UBA and SH3, retains this ability [14,16,35,49]. Hence, direct or indirect binding of TULA-2 to Syk that is dependent on TULA-2 UBA or SH3, albeit possible, is unlikely to be essential for this enzyme–substrate interaction.

Despite the findings indicating that Syk-family PTKs are key substrates of TULA-2, the existence of other protein substrates of TULA-family PTPs cannot be ruled out. For example, TULA-2 has been shown to promote a malignant phenotype of leukemic [63] and breast cancer [64] cells. This effect seems counterintuitive at first, since protein tyrosine phosphorylation, in general, promotes these types of cancer, but TULA-2 in these systems appears to act by dephosphorylating and, thus, down-regulating the E3 ubiquitin-protein ligase Cbl, whose cellular role is to down-regulate PTKs, including those which are oncogenic (reviewed in [58]).

Many other studies suggested that TULA-2 can dephosphorylate Cbl, Src-family PTKs, epithelial growth factor receptor, and other PTKs [14,19,65,66], although the pY-sites targeted by TULA-2 in these proteins have not been identified (reviewed in [32,58]). Some of these sites may conform, fully or partially, to the defined substrate determinants [35]. Others may require TULA-family UBA and/or SH3 domains for substrate interactions or represent sub-optimal targets of TULA-family PTPs that are dephosphorylated due to high local concentrations of substrates and/or phosphatases. Characterization of these sites and identification of novel protein substrates of TULA-family PTPs require further investigation.

Taken together, findings related to the TULA-2 pY-substrates and to the effects of TULA-family proteins on ITAM-/hemITAM-mediated signaling are consistent with the notion of Syk-family PTKs being a central target of this regulatory system. Syk pY346, as well as ZAP-70 pY318, its virtually identical homologue, appear to be the best-known substrate sites of TULA-2 on Syk-family PTKs [24,35,36,49] (see Figure 2 and Figure 3). This site has been shown previously to be critical for the activation of both Syk and ZAP-70 [67,68,69,70,71,72]; hence, its dephosphorylation is expected to exert a regulatory effect on these PTKs. Indeed, a detailed study in a reconstituted system with the GPVI collagen receptor has demonstrated a key role of pY346 dephosphorylation in the down-regulatory effect of TULA-2 on Syk activity and Syk-mediated signaling [49].

However, Syk pY346 is not the only regulatory site located in the interdomain B region of Syk-family PTKs. Two closely spaced pY-sites (Syk pY342 and Syk pY346), as well as the corresponding sites in ZAP-70, are critically involved in the regulation of activity and functions of these PTKs [67,68,69,70,71,72,73,74,75,76,77,78]. Importantly, TULA-2 is also capable of dephospho-rylating Syk pY342, albeit less actively than it dephosphorylates Syk pY346 [49] (see Figure 3). This two-site regulatory circuit is further confounded by the finding that phosphorylation, as well as dephosphorylation of either site, is dependent on the other one [49]. Regardless of the exact molecular mechanisms of the interactions between these two sites in their phosphorylation/dephosphorylation and in their effects on PTK activity, they appear to represent a central element of the mechanism through which TULA-family PTPs—at least TULA-2—exert their regulatory effect on receptor-mediated signaling.

An additional possible element of the regulatory mechanism by which TULA-2 modulates Syk activity is the Syk pY317 site, clearly exhibiting positive substrate specificity determinants (see Figure 3), and whose level of phosphorylation is affected by TULA-2 in activated platelets [35]. In most cell types, Syk pY317 negatively regulates Syk activity by providing a docking point for Cbl, an E3 ubiquitin-protein ligase, which ubiquitylates phosphorylated Syk, targeting it for degradation, thus down-regulating the amount of its active form [60,61,62]. (In platelets, the Cbl–Syk interaction results in ubiquitylation without degradation, which is also inhibitory for Syk [79].) Therefore, TULA-2 appears to exert not only a down-regulatory effect on Syk by dephosphorylating pY346 (and, to some extent, pY342), but also an up-regulatory effect by dephosphorylating pY317, a site of negative regulation. Notably, the TULA-2-mediated dephosphorylation of Syk pY317 is likely to be specific for this member of the Syk family, because ZAP-70 pY290, which corresponds to Syk pY317 and likewise acts as a Cbl-dependent negative regulatory site [80,81], shows very limited similarity to Syk pY317 and weaker specificity determinants as a TULA-2 substrate (see Figure 3). This difference might underlie the differential effects of TULA PTPs—at least of TULA-2—on the two Syk-family PTKs, thus rendering this regulatory circuit even more complex and urging further investigation.

## 8. PTP-Independent Regulatory Effects of TULA-Family Proteins

In addition to well-characterized PTP-mediated effects, TULA-family members exert some biologically significant effects that are mediated by their interactive domains and may likely be independent of PTP activity. For example, both TULA proteins have been shown to reduce production of HIV-1 by inhibiting the late steps of its life cycle in a UBA-dependent fashion. This effect appears to be mediated by binding of TULA proteins to ABCE-1, a host factor of HIV-1 assembly [15]. Also, TULA-1 has been shown to bind to the apoptosis-inducing factor (AIF), a protein inducing caspase-independent cell death under stress conditions, and to facilitate T-cell death in a UBA- and SH3-dependent fashion [13].

Furthermore, TULA-2 has been shown to form a complex with the mitotic kinesin-like protein 2 and Aurora B, a protein kinase that regulates chromosomal segregation. The recruitment of Aurora B, which appears to be mediated by the interactions between its ubiquitin residues and TULA-2 UBA domain, induces anaphase [12]. The well-established binding of TULA-family proteins to Cbl [4,5] might also contribute to the regulation of microtubules, since Cbl has been shown to exert a significant effect on these structures [82].

Findings suggesting that TULA-family proteins play a key role in the biological processes that take place outside the immune/hematological compartment and might be unrelated to ITAM-mediated signaling continue accumulating. Thus, TULA-1 has been implicated in the regulation of human kidney development; in this process, TULA-1 may interact with AIF-related proteins [83]. Another example of an unexpected putative lymphoid-independent function of TULA-family proteins is provided by a strong and specific increase in the level of TULA-2 in mouse brain during the consolidation phase of conditioning [84].

These findings suggest that both family members exert some PTP-unrelated effects, but this ability may be especially important for TULA-1. Although TULA-1 appears to be capable of acting through dephosphorylation of ZAP-70 [24,36], a drastic difference in activity between the two family members, and the established ability of TULA-2 alone to regulate ITAM- and Syk-mediated activation of many cell types, suggests the existence of ITAM-/Syk-independent regulatory mechanisms specific for TULA-1. This notion is also supported by the fact that the TULA-1-encoding gene is responsible for multiple and very diverse autoimmune conditions [85,86,87,88,89,90,91,92,93,94,95,96], while TULA-2 appears to be involved in a single condition only [97,98] (reviewed in [57,58]).

The effect of TULA-1 in type-1 diabetes may be mediated by down-regulation of NF-κB, linked to the interactions of TULA-1 SH3 with NEMO and TAK1, key elements of NF-κB signaling in T cells, which are known to be poly-ubiquitylated, as well as to the interactions of TULA-1 UBA and poly-ubiquitin chains [9]. The down-regulatory effect of TULA-1 on NF-κB signaling results in the suppression of IL-2 production by activated T cells, which appears to underlie the biological/pathological effects of TULA-1 type-1 diabetes risk alleles [9,99]. Another SH3-based molecular mechanism by which TULA-1 may affect T-cell activation is the down-regulatory effect of TULA-1 on the total cellular level of TCR components and the surface level of TCR complexes. This effect appears to depend on the interactions of TULA-1 with multiple proteins, including dynamin and Cbl-b, and, at least in part, on the internalization of TCR from the T-cell surface following its ligand engagement [42].

## 9. Targeting TULA-Family Proteins as a Possible Therapeutic Tool

Considering strong effects of TULA proteins on key biological and pathological processes, their targeting appears to represent a promising therapeutic tool. One may speculate that TULA-1 can be targeted to treat autoimmunity and/or fight infections, while TULA-2 can be targeted to treat hemostasis-/thrombosis-related conditions. However, this area is not extensively studied, and some key issues remain unclear. Thus, the specificity of potential targeting effects is not obvious, because both family members are involved in the regulation of immune responses [36,37,100], and because processes other than immunity and hemostasis/thrombosis are affected by them [21,83,84]. Furthermore, TULA proteins are likely to carry out not only PTP-dependent, but also PTP-independent functions (see above). This feature rules out pharmacological PTP inhibitors, however potent and specific, as a universal tool for targeting TULA proteins, making it necessary to modulate their expression.

Several TULA-2 inhibitors and even their classes have been identified, but none of them are ready for the immediate therapeutic use, due to insufficient potency and/or specificity [34,101,102], thus requiring additional studies in this direction. The possibility of using modulation of TULA-2 expression to affect pathological processes has been shown in a mouse model of heparin-induced thrombocytopenia (HIT); anti-miR-148a increases the level of TULA-2 and protects mice from HIT [61]. Importantly, targeting TULA-2 expression may both facilitate and reduce TULA-2 function by using anti-miR or RNAi reagents, while no pharmacological activators of TULA-2 have been identified to enhance its function. It should also be noted that targeting of TULA-2 has been studied much better than that of TULA-1.

## 10. The Comparative Biochemistry Approach for Revealing the Biological Functions of the TULA Family

Together, the findings discussed above strongly suggest that the TULA-family proteins play a key regulatory role in many disparate biological processes. Although most of the discussed studies focus on T lymphocytes and, more recently, on immune cells of myeloid nature and platelets, this emphasis may, at least in part, be due to the circumstances of the initial characterization of these proteins and the interests of the researchers involved. The role of the TULA family in biological regulation may be substantially broader than our current knowledge suggests. Several other questions still remain to be answered despite the extensive studies of the TULA family conducted over the last two decades. Thus, the reason for employing two family members in signaling regulation, which is especially evident in T lymphocytes, is not obvious, since TULA-2 alone can regulate receptor signaling in other cell types. Can this be explained by differences in the regulatory mechanisms of the individual Syk-family members, thus requiring different sets of TULA-family regulators? It also remains enigmatic how the overlapping functions of the two TULA-family members in T cells relate to the significant difference in their PTP activity. It is especially puzzling, considering that a low-activity member appears to be involved in a large panel of very diverse autoimmune conditions, while only a single autoimmune disorder has been found to be linked to a high-activity member.

### 10.1. TULA-like Genes of Invertebrates

**Figure 4:**
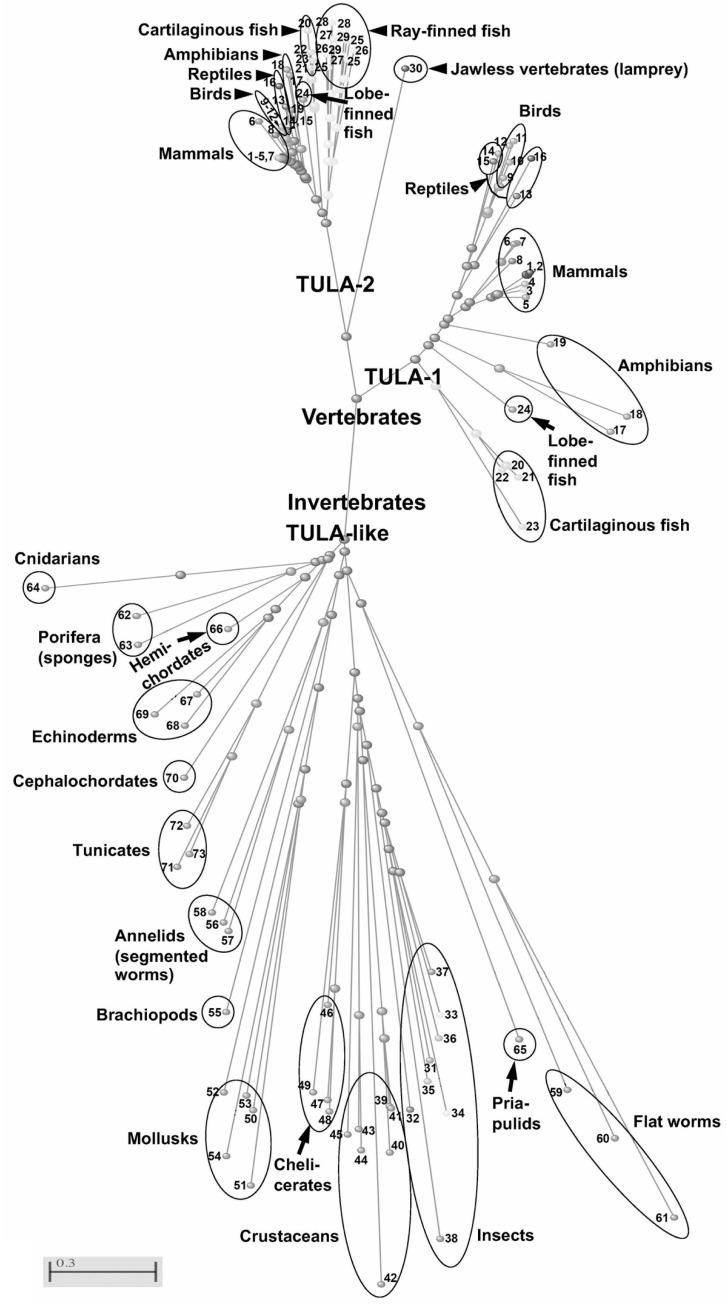
Evolutionary relationships between TULA-family proteins. The analysis was carried out using the Constraint–based Multiple Protein Alignment Tool [103] with the default alignment parameters. The tree (radial) was built using neighbor-joining method with the evolutionary distances calculated according to Grishin [104]. All sequences have been obtained from the Protein database maintained by the National Library of Medicine, National Center for Biotechnology Information. The TULA-family proteins have been selected for analysis from multiple species representing both vertebrates and invertebrates, which are marked by numbers. Relevant taxonomic groups are indicated. The locus designations of the proteins analyzed are given below. When both TULA-1 and TULA-2 have been analyzed, the locus number for TULA-1 precedes that for TULA-2 in the following list. Placental mammals: human (#1, *Homo sapiens*: NP_061834.1 and NP_116262.2), chimpanzee (#2, *Pan troglodytes*: XP_016794065.1 and XP_508828.2), cat domestic (#3, *Felis catus*: XP_003991416.1 and XP_003992499.1), cow (#4, *Bos taurus*: NP_001015599.1 and NP_001193057.1), and house mouse (#5, *Mus musculus*: NP_808491.2 and NP_789830.1). Marsupials: gray short-tailed opossum (#6, *Monodelphis domestica*; XP_007493239.1 and XP_016289274.1), and koala (#7, *Phascolarctos cinereus*: XP_020829536.1 and XP_020840539.1). Monotremes: platypus (#8, *Ornithorhynchus anatinus*: XP_039770616.1 and XP_028931626.1). Birds: chicken (#9, *Gallus gallus*: XP_004934641.1 and XP_015153661.1), turkey (#10, *Meleagris gallopavo*: XP_010722714.1 and XP_031412703.1), hooded crow (#11, *Corvus cornix cornix*: XP_019138194.1 and XP_039420798.1), and common cuckoo (#12, *Cuculus canorus*: XP_009559175.1 and XP_009555828.1). Reptiles: green anole (#13, *Anolis carolinensis*: XP_008105681.1 and XP_016850210.1), American alligator (#14, *Alligator mississippiensis*: XP_019342726.1 and XP_006275146.1), painted turtle (#15, *Chrysemys picta bellii*: XP_005303759.1 and XP_005303797.1), and brown-spotted pit viper (#16, *Protobothrops mucrosquamatus*: XP_015677410.1 and XP_015676892.1). Amphibians: African clawed frog (#17, *Xenopus laevis*: XP_041439474.1 and XP_018081241.1), common toad (#18, *Bufo bufo*: XP_040278639.1 and XP_040287247.1), and Iberian ribbed newt (#19, *Pleurodeles waltl*: KAJ1112034.1 and KAJ1175306.1). Fish—sharks: whale shark (#20, *Rhincodon typus*: XP_048459103.1 and XP_048471549.1), zebra shark (#21, *Stegostoma fasciatum*: XP_048396616.1 and XP_048417962.1), smaller spotted catshark (#22, *Scyliorhinus canicular*: XP_038657220.1 and XP_038634591.1), smalltooth sawfish (#23, *Pristis pectinate*: XP_051870461.1 and XP_051895694.1). Fish—lobe-finned fish (Sarcopterygii): coelacanth (#24, *Latimeria chalumnae*: XP_014354497.1 partial and XP_005987327.1). Fish—ray-finned fishes (Actinopterygii; the proteins analyzed for ray-finned species are TULA-2a and TULA-2b listed first and second, respectively, for each species, except for the Atlantic salmon, where TULA-2a, TULA-2a’ and TULA-2b are listed): Atlantic salmon (#25, *Salmo salar*: XP_014069250.1, XP_014017956.1 and XP_014051313.1), zebrafish (#26, *Danio rerio*: NP_001122227.1 and XP_001923885.2), Nile tilapia (#27, *Oreochromis niloticus*: XP_013121081.1 and XP_003446846.1), moonfish (#28, *Xiphophorus maculatus*: XP_023207530.1 and XP_005802954.1), and mudskipper (#29, *Boleophthalmus pectinirostris*: XP_020782492.1 and XP_020778647.1). For a detailed tree of fish TULA sequences, see Figure 5. Jawless vertebrates: sea lamprey (#30, *Petromyzon marinus*: XP_032820445.1; only TULA-2 is listed, no TULA-1 has been found). Insects: yellow fever mosquito (#31, *Aedes aegypti*: XP_021693348.1), honeybee (#32, *Apis mellifera*: XP_394838.2), powderpost termite (#33, *Cryptotermes secundus*: XP_023711760.1), fruit fly (#34, *Drosophila melanogaster*: NP_651202.1), cat flea (#35, *Ctenocephalides felis*: XP_026468601.1), red flour beetle (#36, *Tribolium castaneum*: KYB26555.1), desert locust (#37, *Schistocerca gregaria*: XP_049863752.1), and fall armyworm (#38, *Spodoptera frugiperda*: XP_050561310.1). Crustacean: Pacific white shrimp (#39, *Penaeus vannamei*: XP_027239151.1), snow crab (#40, *Chionoecetes opilio*: KAG0711382.1), Louisiana crawfish (#41, *Procambarus clarkii*: XP_045594025.1), Hyalella Azteca (#42, *Hyalella azteca*: XP_047735455.1), striped barnacle (#43, *Amphibalanus amphitrite*: XP_043215535.1), goose barnacle (#44, *Pollicipes pollicipes*: XP_037086318.1), and water flea (#45, *Daphnia pulex*: XP_046453513.1). Chelicerata: deer tick (#46, *Ixodes scapularis*: XP_029842566.1), Western predatory mite (#47, *Galendromus occidentalis*: XP_018494053.1), honeybee mite (#48, *Varroa destructor*: XP_022654098.1), and horse-shoe crab (#49, *Limulus polyphemus*: XP_022253956.1). Mollusks: Virginia oyster (#50, *Crassostrea virginica*: XP_022341161.1), California mussel (#51, *Mytilus californianus*: XP_052075640.1), California sea hare (#52, *Aplysia californica*: XP_005099706.1), red abalone (#53, *Haliotis rufescens*: XP_048237131.1), and California two-spot octopus (#54, *Octopus bimaculoides*: XP_052830112.1). Brachiopods: *Lingula anatina* (#55, XP_013419245.1). Segmented worms (annelids): marine polychaeta *Capitella teleta* (#56, ELU07750.1), leach (#57, *Helobdella robusta*: XP_009015730.1), and tubeworm (#58, *Owenia fusiformis*: CAH1800724.1). Flat worms: dog tapeworm (#59, *Echinococcus granulosus*: KAH9282803.1), Chinese liver fluke (#60, *Clonorchis sinensis*: KAG5444987.1), and blood-fluke (#61, *Schistosoma intercalatum*: CAH8519421.1). Sponges: *Amphimedon queenslandica* (#62, XP_019855686.1), and *Oopsacas minuta* (#63, KAI6655504.1). Cnidarians: fresh-water polyp (#64, *Hydra vulgaris*: XP_047130900.1). Priapulids: cactus worm (#65, *Priapulus caudatus*: XP_014678843.1). Hemichordates: acorn worm (#66, *Saccoglossus kowalevskii*: XP_006821603.1). Echinoderms: Crown-of-thorns starfish (#67, *Acanthaster planci*: XP_022099247.1), purple sea urchin (#68, *Strongylocentrotus purpuratus*: XP_030834281.1), and *Anneissia japonica* (#69, XP_033119264.1). Cephalochordates: Belcher’s lancelet (#70, *Branchiostoma belcheri*: XP_019620650.1). Tunicates: vase tunicate (#71, *Ciona intestinales*: XP_026692047.1), rough sea squirt (#72, *Styela clava*: XP_039248084.1), and *Phallusia mammillata* (#73, CAB3267428.1).

One possible independent approach that may facilitate further understanding of this family and its biological functions rests in inquiring into the issues related to its origin. This approach may help us to evaluate this family’s ‘reason for being’ by conducting the comparative analysis of TULA proteins in diverse species and taxa. A close look at the TULA family tree reveals the existence of a TULA-like protein-encoding gene in almost all the reviewed invertebrate taxa. These taxa are extremely diverse; hence, it appears that the presence of a TULA-like protein is typical for invertebrates (Figure 4).

There is one notable exception to this rule. Most TULA-homologous sequences annotated in roundworm genomes lack an extensive N-terminal portion characteristic of all other TULA-type vertebrate and invertebrate genes. Several annotated roundworm genes possess an N-terminal part, but it is quite dissimilar to the TULA genes of all other species examined. Furthermore, the key histidine residue of mammalian TULA-family PTPs (His-380 of mouse TULA-2), which is conserved in their vertebrate and invertebrate homologues, is not fully conserved in roundworms. However, it cannot be ruled out that the observed anomalies in the annotated roundworm sequences are due to the failure of the prediction algorithms in this case.

### 10.2. TULA-Family in Vertebrates: Conserved and Unique Features

The origin of the TULA family is clearly associated with the origin of vertebrates; all vertebrates have genes unambiguously recognized as encoding either TULA-1 (UBASH3A) or TULA-2 (UBASH3B). Such diverse vertebrate taxa as mammals and other tetrapods, lobe-finned fish (latimeria, which is considered a ‘living fossil’), and cartilaginous fish (sharks) possess both genes, thus exhibiting a conserved family structure. These findings are consistent with a duplication of the TULA-family ancestral gene at the root of the vertebrate tree, likely as a result of whole-genome duplication [105].

**Figure 5:**
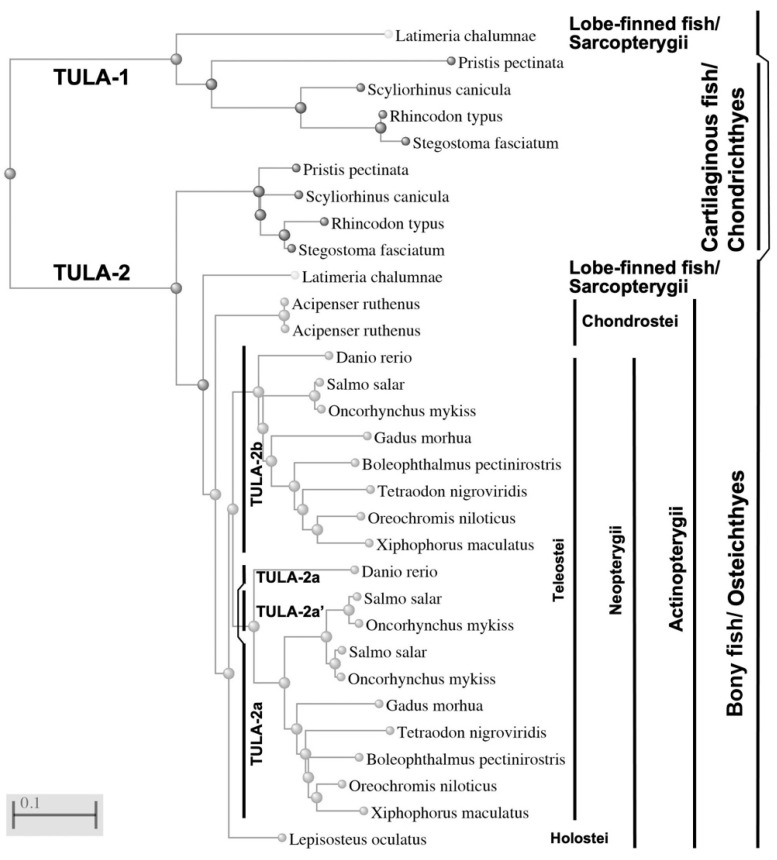
Evolutionary relationships between fish TULA proteins. The TULA-family proteins have been selected for analysis from major fish taxonomic groups and analyzed as shown in Figure 4. The types of TULA-family proteins and relevant taxa are indicated on the diagram. The locus designations of the proteins analyzed are listed below. The structure of the TULA family in cartilaginous (sharks) and lobe-finned (coelacanth) fish is conserved; hence, two proteins listed for these species are TULA-1 (first) and TULA-2 (second). In teleosts, except for salmonids, TULA-2a and TULA-2b are listed as the first and second loci. For salmonids, which have a three-gene TULA family, TULA-2a, TULA-2a’, and TULA-2b are listed in this order. Only a single TULA-2 gene is present in the spotted gar. Two poorly differentiated TULA-2-derived genes that cannot be classified as TULA-2a and TULA-2b are present in the sterlet. Spotted gar (*Lepisosteus oculatus*: XP_006642212.1). Sterlet (*Acipenser ruthenus*: XP_033851636.1 and XP_034768603.1). Sharks: whale shark (*Rhincodon typus*: XP_048459103.1 and XP_048471549.1), zebra shark (*Stegostoma fasciatum*: XP_048396616.1 and XP_048417962.1), smaller spotted catshark (*Scyliorhinus canicular*: XP_038657220.1 and XP_038634591.1), and smalltooth sawfish (*Pristis pectinate*: XP_051870461.1 and XP_051895694.1). Coelacanth (*Latimeria chalumnae*: XP_014354497.1 partial and XP_005987327.1). Teleosts: Atlantic salmon (*Salmo salar*: XP_014069250.1, XP_014017956.1 and XP_014051313.1), rainbow trout (*Oncorhynchus mykiss*: XP_021437951.2, XP_021442713.2, and XP_036789231.1), Atlantic cod (*Gadus morhua*: XP_030236513.1 and XP_030216786.1), spotted green pufferfish (*Tetraodon nigroviridis*: CAG06470.1 and CAG02883.1), zebrafish (*Danio rerio*: NP_001122227.1 and XP_001923885.2), Nile tilapia (*Oreochromis niloticus*: XP_013121081.1 and XP_003446846.1), moonfish (*Xiphophorus maculatus*: XP_023207530.1 and XP_005802954.1), and mudskipper (*Boleophthalmus pectinirostris*: XP_020782492.1 and XP_020778647.1).

Whereas jawless vertebrates, such as lamprey, have a single family member, it is substantially dissimilar to the invertebrate TULA-like protein and clearly belongs to the TULA-2 branch of the family, suggesting that, following a duplication of the invertebrate gene, one family member has been lost in this lineage. A scenario similar to lampreys is also apparent for jawed vertebrates; while both cartilaginous fish, which are considered to be a taxon closest to the vertebrate ancestral form, and lobe-finned fish, which represent a sister group of tetrapods, have a two-member TULA family similar to that in mammals, no species among the examined ray-finned fish has a TULA-1-encoding gene, arguing that TULA-1 has been lost at the root of the ray-finned fish tree (Figure 5). Consistent with this notion, the spotted gar *Lepisosteus oculatus* of the ancient Holostei class has a single TULA-2 gene. The sterlet *Acipenser ruthenus* (class Chondrostei) has two genes which encode TULA-2-related proteins, but their protein products are different by a single amino acid, suggesting a gene duplication that has not been followed by differentiation yet.

In contrast, the teleosts demonstrate a unique TULA family of at least two members, which have been termed TULA-2a and TULA-2b [58]. Both teleost family members are clearly related to TULA-2, not to TULA-1, but exhibit substantial intraspecies differences. Thus, TULA-2a and TULA-2b demonstrate only 75% and 73% identity in *Danio rerio* and *Xiphophorus maculatus*, two common fish experimental models, respectively; these differences are very close to those between human and shark TULA-2 (75–77% identity). Furthermore, the salmonids also have TULA-2a’, a TULA-2a-like gene, arguing that an additional duplication occurred in this branch (see Figure 5). Therefore, the loss of TULA-1 in the ray-finned fish is followed by TULA-2 duplications and ensuing differentiation, resulting in the development of the unique teleost TULA family, which reaches its apex in salmonids.

TULA-2 is extremely conserved throughout vertebrates (the sequence identities between human TULA-2 and TULA-2 from mice, chicken, and coelacanth are 98, 90, and 82%, respectively), whereas TULA-1 is significantly less conserved (78, 63, and 52%, respectively). The presence of TULA-family members in all vertebrates, the highly conserved nature of this family, and, in particular, the highly conserved sequence of TULA-2 within vertebrates is consistent with the idea of critical involvement of this family in the central biological functions of vertebrates that distinguish them from invertebrates.

### 10.3. Invertebrate Functions of TULA-like Proteins

Whereas vertebrate TULA-family proteins have been studied extensively in human and mouse systems, little is known about invertebrate TULA-like proteins. Some arthropod TULA-like proteins have been characterized as ecdysteroid-phosphate phosphatases (EPPs) [106,107], which release ecdysteroids from ecdysteroid-phosphates, thus regulating the arthropod embryonic development [108,109]. The existence of a link between EPP and embryonic development is supported by the pattern of changes in EPP expression and EPP activity, which is nearly parallel to that of change in the amount of free ecdysteroids in the developing egg of the silkworm *Bombyx mori* [108]. Furthermore, the role of EPP in the water flea *Daphnia magna* has been shown using RNAi-depletion of EPP message in the developing egg of this crustacean; its development is blocked by an EPP-targeting RNAi [109].

However, it is unclear whether ecdysteroid-phosphates represent major biologically relevant substrates of the EPPs, since mammalian TULA-2 can also dephosphorylate ecdysteroid-phosphates, while EPP can dephosphorylate pY-peptides and pY-proteins [106,107]. Furthermore, hydrolysis of ecdysteroid-phosphates is unlikely to be a universal invertebrate function of TULA-like proteins, since the great majority of invertebrates lack ecdysteroids.

While invertebrate TULA-like proteins cannot be identified exactly as either TULA-1 or TULA-2, representing a lineage clearly separate from both vertebrate family members (see Figure 4), sequence analysis indicates a very high degree of conservation for all key catalytic residues between vertebrate TULA-family proteins and invertebrate TULA-like proteins, in agreement with the idea that the latter are active PTPs. This idea is further supported by the finding revealing that the structure of the silkworm EPP catalytic domain is similar to that of mammalian TULA proteins [106,107].

The conserved nature of invertebrate TULA-like protein sequences suggests that these proteins are conserved functionally as well. Considering that the differences in anatomy and physiology between invertebrate taxa are very dramatic, it is reasonable to hypothesize that the common regulatory targets of TULA-like proteins play a role in the fundamental biochemical processes which are universal for all metazoans.

### 10.4. TULA-Family Proteins Function in Vertebrate-Specific Systems

Whereas invertebrate species have a single gene encoding for a TULA-like protein, the two-member TULA family is typical for vertebrates (see Figure 4). Thus, the origin of the TULA family correlates with the emergence of novel organ systems and novel functions at the cellular level, including the two functions in which this family has already been shown to play a key role: adaptive immunity mediated by lymphocytes (TULA-1 and TULA-2) and hemostasis in the closed blood circulation system requiring thrombocytes/platelets (TULA-2). Importantly, both family members are involved in the regulation of adaptive immunity, where their functional overlap has been shown originally [3,19], and where their specific effects have been demonstrated [36], including differential contributions to autoimmune diseases [57,58].

The effect of the TULA family on cells which mediate innate immune responses has also been shown [33,41,100], but the partial contributions of the family members are less understood in these systems. The only process where these contributions have been clearly delineated to date is the IFN-γ-induced nitric oxide production by bone marrow-derived monocytes [41]. The involvement of both TULA-family members in innate immunity might seem inconsistent with the idea of the origin of this family being linked to the emergence of adaptive immunity, since the presence of innate immunity and/or immune-type recognition is evident in various invertebrate taxa [110,111,112,113], which possess a single TULA-like gene. However, it should be noted that innate immunity in vertebrates is more complex than that in invertebrates since many functions of the innate immune system in vertebrates are regulated by and dependent on adaptive immunity. The involvement of both family members in vertebrate innate immune responses may be linked to this essential interaction.

Another key regulatory function of the TULA family in vertebrates has been demonstrated in platelets, whose major role is to constantly survey the integrity of the blood circulatory system and to mediate hemostatic responses by forming an aggregate, which seals off a damaged area in a blood vessel [16,35,49] (reviewed in [53]). Therefore, these anucleate cells, together with nucleated thrombocytes, which are present in vertebrates other than mammals, play a role unique for vertebrates, since no invertebrate possesses a classically defined closed circulatory system [114]. The platelet-centered functions of the family require only TULA-2 [49]. This result is not inconsistent with the notion that the origin of the TULA family is critically linked to the emergence of specific vertebrate systems and cellular functions, because TULA-2 is clearly distinct from invertebrate TULA-like proteins, which belong to a separate and very diverse lineage, and, hence, may be distinct functionally as well.

### 10.5. Atypical Single-TULA Taxa and the Unique TULA Family of the Teleost Fish

Important in this regard are the exceptions from the general two-member vertebrate pattern, such as lampreys, Chondrostei (sturgeons), and Holostei (gars). (Although, strictly speaking, the sterlet (Chondrostei) has two TULA-2-type genes, its protein products differ by a single amino acid substitution, which is conserved, thus rendering the difference between protein family members in this group negligible.) Both fish and lampreys possess the closed circulation system equipped with thrombocytes, which is characteristic of all vertebrates [115,116,117,118,119,120,121,122,123]. Likewise, they possess adaptive immunity, which is also a common feature of all vertebrates, albeit unique adaptive immunity traits exist in each of these groups [113,123,124,125,126,127,128,129]. Thus, adaptive immunity of jawless vertebrates (lampreys) is distinct from that of jawed vertebrates, being built on an antigen receptor very different than that characteristic of jawed vertebrates. However, the adaptive immune system of lampreys includes lymphoid cell lineages that closely resemble vertebrate T and B cells [124,125,126,127,128,129]. Therefore, if thrombocytes and lymphocytes of Chondrostei and Holostei fish and jawless vertebrates are regulated by TULA-2, as is expected, they are regulated by TULA-2 alone without any contribution from TULA-1, suggesting that the overlapping effects of two family members observed in mammalian T cells might not be fundamentally essential for the regulation of lymphoid cells by TULA proteins. However, a fundamental difference in responses and/or regulatory mechanisms between mammalian T cells and lymphocytes of ancient fish taxa and lamprey cannot be ruled out either.

Of further interest is the unique TULA-family structure in teleosts; despite the apparent adaptation of lampreys and ancient fish taxa to the loss of TULA-1, a new form of this family is restored in teleosts as an apparent result of UBASH3B duplications. Most teleosts have two members of this unique family—TULA-2a and TULA-2b, while salmonids have three—TULA-2a, TULA-2a’, and TULA-2b. The difference between TULA-2a and TULA-2b within species exceeds that between TULA-2 of some very distant species, but is still less extensive than the difference between TULA-1 and TULA-2 (see Section 10.2 above). Therefore, it remains to be determined whether the teleost TULA-family members can exert specific regulatory effects similar to those exerted by mammalian TULA-1 and TULA-2.

### 10.6. Functional Crosstalk between TULA- and Syk-like Proteins throughout Metazoan Taxa

Most TULA-family functions in both lymphocytes and platelets that have been studied in detail are critically dependent on ITAM-/hemITAM-mediated signaling through ZAP-70 and Syk, which are strongly regulated by TULA-family proteins, especially TULA-2 (see above). Therefore, a close look at a possible link between Syk-family PTKs and TULA-family PTPs in various taxa is warranted. The vertebrate Syk family is represented by Syk and ZAP-70 genes; both PTKs bind to phosphorylated ITAM-bearing receptors and play a key role in propagating signals from such receptors. However, ZAP-70 is expressed in T and NK cells, while Syk is expressed more ubiquitously; hence, ZAP-70 is an essential PTK of T-cell signaling, while Syk plays this role in B cells, platelets, and many myeloid cells [44,53,130,131,132]. Although some structural and functional differences between ZAP-70 and Syk have been revealed [67,72,133], these PTKs are extremely homologous within their conserved functional regions, including the Syk pY346/ZAP-70 pY318 regulatory site, a major known target of TULA-2 in cellular proteins. This finding is consistent with the highly conserved nature of the vertebrate TULA family.

In contrast to vertebrates, invertebrates have only a single Syk-like gene. In addition to this gene, the HTK16 gene (also termed SHARK), which possesses extensive regions of Syk homology but also an ankyrin-repeat region, is present in invertebrates and is unique to them, being dissimilar to any vertebrate PTK. These observations may lead to the hypothesis that the invertebrate PTKs homologous to vertebrate Syk and ZAP-70 are targeted by the invertebrate TULA-like PTPs. However, the major TULA-2 substrate site of mammalian Syk-family PTKs homologous to mouse Syk pY346 is not conserved in either invertebrate Syk or SHARK. Furthermore, not all invertebrate Syk-homologous PTKs possess tyrosine residues in the interdomain B region, which are targeted by TULA-2 in mammalian Syk-family PTKs. Therefore, if invertebrate TULA-like proteins act indeed as PTPs, their targets are likely to be different from those of mammalian TULA-2.

### 10.7. Issues That May Be Addressed by Employing the Comparative Analysis of TULA Proteins in Various Taxa

The current considerable knowledge of the TULA family in mammals and a few studies of arthropod TULA-like proteins, together with the genome sequencing data on TULA in various taxa, encourage us to address several key issues related to the biological role of this family by using comparative analysis of TULA protein functions in various distinct metazoan taxa. Using this approach, we should be able to answer the question of whether the regulation of immunity and hemostasis is indeed a common function in all vertebrate taxa, as was hypothesized. Perhaps, comparison of dissimilar vertebrate taxa will allow us to identify the role(s) of TULA-family proteins outside of immunity or hemostasis.

We may also ask whether the functions of this family are related to the regulation of Syk-family PTKs in all vertebrate taxa. What are the other dephosphorylation targets at a molecular level? Are these putative non-Syk targets common for all vertebrate taxa or are they taxon-specific? This approach would complement any systematic unbiased substrate screening conducted in mammalian systems.

Are the PTP-independent functions of TULA proteins, including an adaptor function, common or specific for various taxa? Do other regulatory systems, such as NF-κB signaling, play a role in the common vertebrate effects of TULA proteins? The comparative analysis of TULA-family functions in various groups of vertebrates is also expected to help us in determining the specific contributions of PTP-dependent and -independent effects of TULA proteins to the overall biological role of this family.

Does the regulation of immunity and/or hemostasis represent a major TULA function in the vertebrate groups demonstrating deviations from the classical conserved family structure? What is the mechanism by which a single family member mediates the regulatory functions in such groups? Are such differences in TULA functions, if observed, underlined by substantial differences of T cell and/or thrombocytes between mammals and ‘single-member taxa’, or do these taxa demonstrate additional molecular mechanisms, substituting for the absence of TULA-1? Finding these mechanisms would considerably advance our understanding of the TULA-1 function.

What is the extent of TULA-family members’ differentiation in teleosts? How distinct are individual members of the unique teleost TULA family in the regulation of immunity and hemostasis? Does a pair of TULA-2-derived genes (or a trio of them in salmonids) function as the TULA-2/TULA-1 pair in mammals, or does it substitute for TULA-2 only? Comparison of this unique family to the typical one is likely to reveal the potential as well as the limits of TULA-family member differentiation.

What are the functions of invertebrate TULA-like proteins? Do they act as PTPs by dephosphorylating regulatory targets? Are these functions related to invertebrate Syk-like PTKs? If so, what is the TULA-like PTPs’ target at the level of specific pY-sites? If not, what are the invertebrate substrates of TULA-like PTPs? Are these targets common for all invertebrates or specific in different taxa? Do the invertebrate systems/functions regulated by TULA-like proteins and the vertebrate systems regulated by the TULA family appear to be connected by some evolutionary links, or are these systems totally distinct and unrelated?

Addressing these issues by analyzing diverse metazoan taxa should be very beneficial to our understanding of the functions of TULA-family proteins in mammals, a vertebrate group naturally attracting our utmost attention. Perhaps by taking a look at the broader picture, we will better understand the TULA family ‘reason for being’, and this, in turn, will assist us in fine-tuning our research program. It should be noted that this approach has received very limited attention up to date and, thus, requires a considerable amount of work focused on organisms never analyzed before in connection to the functions of TULA proteins.

## Data Availability

Not applicable.

## References

[1] Wattenhofer M., Shibuya K., Kudoh J., Lyle R., Michaud J., Rossier C., Kawasaki K., Asakawa S., Minoshima S., Berry A. (2001). Isolation and characterization of the UBASH3A gene on 21q22.3 encoding a potential nuclear protein with a novel combination of domains. Hum. Genet..

[2] Carpino N., Kobayashi R., Zang H., Takahashi Y., Jou S.T., Feng J., Nakajima H., Ihle J.N. (2002). Identification, cDNA cloning, and targeted deletion of p70, a novel, ubiquitously expressed SH3 domain-containing protein. Mol. Cell. Biol..

[3] Carpino N., Turner S., Mekala D., Takahashi Y., Zang H., Geiger T.L., Doherty P., Ihle J.N. (2004). Regulation of ZAP-70 activation and TCR signaling by two related proteins, Sts-1 and Sts-2. Immunity.

[4] Feshchenko E.A., Smirnova E.V., Swaminathan G., Teckchandani A.M., Agrawal R., Band H., Zhang X., Annan R.S., Carr S.A., Tsygankov A.Y. (2004). TULA: An SH3- and UBA-containing protein that binds to c-Cbl and ubiquitin. Oncogene.

[5] Kowanetz K., Crosetto N., Haglund K., Schmidt M.H., Heldin C.H., Dikic I. (2004). Suppressors of T-cell receptor signaling Sts-1 and Sts-2 bind to Cbl and inhibit endocytosis of receptor tyrosine kinases. J. Biol. Chem..

[6] Tsygankov A. (2008). Multi-domain STS/TULA protein are novel cellular regulators. IUBMB Life.

[7] Tsygankov A.Y. (2009). TULA-family proteins: An odd couple. Cell. Mol. Life Sci..

[8] Bertelsen V., Breen K., Sandvig K., Stang E., Madshus I.H. (2007). The Cbl-interacting protein TULA inhibits dynamin-dependent endocytosis. Exp. Cell Res..

[9] Ge Y., Paisie T.K., Newman J.R.B., McIntyre L.M., Concannon P. (2017). UBASH3A Mediates Risk for Type 1 Diabetes Through Inhibition of T-Cell Receptor-Induced NF-kappaB Signaling. Diabetes.

[10] Hoeller D., Crosetto N., Blagoev B., Raiborg C., Tikkanen R., Wagner S., Kowanetz K., Breitling R., Mann M., Stenmark H. (2006). Regulation of ubiquitin-binding proteins by monoubiquitination. Nat. Cell Biol..

[11] Carpino N., Chen Y., Nassar N., Oh H.W. (2009). The Sts proteins target tyrosine phosphorylated, ubiquitinated proteins within TCR signaling pathways. Mol. Immunol..

[12] Krupina K., Kleiss C., Metzger T., Fournane S., Schmucker S., Hofmann K., Fischer B., Paul N., Porter I.M., Raffelsberger W. (2016). Ubiquitin Receptor Protein UBASH3B Drives Aurora B Recruitment to Mitotic Microtubules. Dev. Cell.

[13] Collingwood T.S., Smirnova E.V., Bogush M., Carpino N., Annan R.S., Tsygankov A.Y. (2007). T-cell ubiquitin ligand affects cell death through a functional interaction with apoptosis-inducing factor, a key factor of caspase-independent apoptosis. J. Biol. Chem..

[14] Agrawal R., Carpino N., Tsygankov A. (2008). TULA proteins regulate activity of the protein tyrosine kinase Syk. J. Cell Biochem..

[15] Smirnova E.V., Collingwood T.S., Bisbal C., Tsygankova O.M., Bogush M., Meinkoth J.L., Henderson E.E., Annan R.S., Tsygankov A.Y. (2008). TULA proteins bind to ABCE-1, a host factor of HIV-1 assembly, and inhibit HIV-1 biogenesis in a UBA-dependent fashion. Virology.

[16] Thomas D.H., Getz T.M., Newman T.N., Dangelmaier C.A., Carpino N., Kunapuli S.P., Tsygankov A.Y., Daniel J.L. (2010). A novel histidine tyrosine phosphatase, TULA-2, associates with Syk and negatively regulates GPVI signaling in platelets. Blood.

[17] de Castro R.O., Zhang J., Groves J.R., Barbu E.A., Siraganian R.P. (2012). Once phosphorylated, tyrosines in carboxyl terminus of protein-tyrosine kinase Syk interact with signaling proteins, including TULA-2, a negative regulator of mast cell degranulation. J. Biol. Chem..

[18] van der Meulen T., Swarts S., Fischer W., van der Geer P. (2017). Identification of STS-1 as a novel ShcA-binding protein. Biochem. Biophys. Res. Commun..

[19] Mikhailik A., Ford B., Keller J., Chen Y., Nassar N., Carpino N. (2007). A phosphatase activity of Sts-1 contributes to the suppression of TCR signaling. Mol. Cell.

[20] Chen Y., Jakoncic J., Carpino N., Nassar N. (2009). Structural and functional characterization of the 2H-phosphatase domain of Sts-2 reveals an acid-dependent phosphatase activity. Biochemistry.

[21] Back S.H., Adapala N.S., Barbe M.F., Carpino N.C., Tsygankov A.Y., Sanjay A. (2013). TULA-2, a novel histidine phosphatase, regulates bone remodeling by modulating osteoclast function. Cell. Mol. Life Sci..

[22] Rigden D.J. (2008). The histidine phosphatase superfamily: Structure and function. Biochem. J..

[23] Chen Y., Jakoncic J., Parker K.A., Carpino N., Nassar N. (2009). Structures of the phosphorylated and VO(3)-bound 2H-phosphatase domain of Sts-2. Biochemistry.

[24] San Luis B., Sondgeroth B., Nassar N., Carpino N. (2011). Sts-2 is a phosphatase that negatively regulates zeta-associated protein (ZAP)-70 and T cell receptor signaling pathways. J. Biol. Chem..

[25] Sadatomi D., Tanimura S., Ozaki K., Takeda K. (2013). Atypical protein phosphatases: Emerging players in cellular signaling. Int. J. Mol. Sci..

[26] Tonks N.K. (2013). Protein tyrosine phosphatases--from housekeeping enzymes to master regulators of signal transduction. FEBS J..

[27] Tiganis T., Bennett A.M. (2007). Protein tyrosine phosphatase function: The substrate perspective. Biochem. J..

[28] Kleinman H., Ford B., Keller J., Carpino N., Nassar N. (2006). Crystallization and initial crystal characterization of the C-terminal phosphoglycerate mutase homology domain of Sts-1. Acta Crystallogr. Sect. F Struct. Biol. Cryst. Commun..

[29] Mazumder R., Iyer L.M., Vasudevan S., Aravind L. (2002). Detection of novel members, structure-function analysis and evolutionary classification of the 2H phosphoesterase superfamily. Nucleic Acids Res..

[30] Yin Y., Frank D., Zhou W., Kaur N., French J.B., Carpino N. (2020). An unexpected 2-histidine phosphoesterase activity of suppressor of T-cell receptor signaling protein 1 contributes to the suppression of cell signaling. J. Biol. Chem..

[31] San Luis B., Nassar N., Carpino N. (2013). New insights into the catalytic mechanism of histidine phosphatases revealed by a functionally essential arginine residue within the active site of the Sts phosphatases. Biochem. J..

[32] Tsygankov A.Y. (2020). TULA proteins as signaling regulators. Cell Signal..

[33] Frank D., Naseem S., Russo G.L., Li C., Parashar K., Konopka J.B., Carpino N. (2018). Phagocytes from Mice Lacking the Sts Phosphatases Have an Enhanced Antifungal Response to Candida albicans. MBio.

[34] Zhou W., Yin Y., Weinheimer A.S., Kaur N., Carpino N., French J.B. (2017). Structural and Functional Characterization of the Histidine Phosphatase Domains of Human Sts-1 and Sts-2. Biochemistry.

[35] Chen X., Ren L., Kim S., Carpino N., Daniel J.L., Kunapuli S.P., Tsygankov A.Y., Pei D. (2010). Determination of the substrate specificity of protein-tyrosine phosphatase TULA-2 and identification of Syk as a TULA-2 substrate. J. Biol. Chem..

[36] Newman T.N., Liverani E., Ivanova E., Russo G.L., Carpino N., Ganea D., Safadi F., Kunapuli S.P., Tsygankov A.Y. (2014). Members of the novel UBASH3/STS/TULA family of cellular regulators suppress T-cell-driven inflammatory responses in vivo. Immunol. Cell Biol..

[37] Naseem S., Frank D., Konopka J.B., Carpino N. (2015). Protection from systemic Candida albicans infection by inactivation of the Sts phosphatases. Infect. Immun..

[38] Hofs S., Mogavero S., Hube B. (2016). Interaction of Candida albicans with host cells: Virulence factors, host defense, escape strategies, and the microbiota. J. Microbiol..

[39] Netea M.G., Joosten L.A., van der Meer J.W., Kullberg B.J., van de Veerdonk F.L. (2015). Immune defence against Candida fungal infections. Nat. Rev. Immunol..

[40] Feller L., Khammissa R.A., Chandran R., Altini M., Lemmer J. (2014). Oral candidosis in relation to oral immunity. J. Oral Pathol. Med..

[41] Parashar K., Carpino N. (2020). A role for the Sts phosphatases in negatively regulating IFNgamma-mediated production of nitric oxide in monocytes. Immun. Inflamm. Dis..

[42] Ge Y., Paisie T.K., Chen S., Concannon P. (2019). UBASH3A Regulates the Synthesis and Dynamics of TCR-CD3 Complexes. J. Immunol..

[43] Lim C.K., Hwang W.Y., Aw S.E., Sun L. (2008). Study of gene expression profile during cord blood-associated megakaryopoiesis. Eur. J. Haematol..

[44] Tsygankov A.Y. (2003). Non-receptor protein tyrosine kinases. Front. Biosci..

[45] Watson S.P. (1999). Collagen receptor signaling in platelets and megakaryocytes. Thromb. Haemost..

[46] Clark E.A., Shattil S.J., Brugge J.S. (1994). Regulation of protein tyrosine kinases in platelets. Trends Biochem. Sci..

[47] Zeiler M., Moser M., Mann M. (2014). Copy number analysis of the murine platelet proteome spanning the complete abundance range. Mol. Cell. Proteom..

[48] Burkhart J.M., Vaudel M., Gambaryan S., Radau S., Walter U., Martens L., Geiger J., Sickmann A., Zahedi R.P. (2012). The first comprehensive and quantitative analysis of human platelet protein composition allows the comparative analysis of structural and functional pathways. Blood.

[49] Reppschlager K., Gosselin J., Dangelmaier C.A., Thomas D.H., Carpino N., McKenzie S.E., Kunapuli S.P., Tsygankov A.Y. (2016). TULA-2 Protein Phosphatase Suppresses Activation of Syk through the GPVI Platelet Receptor for Collagen by Dephosphorylating Tyr(P)346, a Regulatory Site of Syk. J. Biol. Chem..

[50] Zhou Y., Abraham S., Andre P., Edelstein L.C., Shaw C.A., Dangelmaier C.A., Tsygankov A.Y., Kunapuli S.P., Bray P.F., McKenzie S.E. (2015). Anti-miR-148a regulates platelet FcgammaRIIA signaling and decreases thrombosis in vivo in mice. Blood.

[51] Zhou Y., Abraham S., Renna S., Edelstein L.C., Dangelmaier C.A., Tsygankov A.Y., Kunapuli S.P., Bray P.F., McKenzie S.E. (2016). TULA-2 (T-Cell Ubiquitin Ligand-2) Inhibits the Platelet Fc Receptor for IgG IIA (FcgammaRIIA) Signaling Pathway and Heparin-Induced Thrombocytopenia in Mice. Arterioscler. Thromb. Vasc. Biol..

[52] Kostyak J.C., Mauri B.R., Dangelmaier C., Patel A., Zhou Y., Eble J.A., Tsygankov A.Y., McKenzie S.E., Kunapuli S.P. (2018). TULA-2 Deficiency Enhances Platelet Functional Responses to CLEC-2 Agonists. TH Open.

[53] Kunapuli S.P., Tsygankov A.Y. (2022). TULA-Family Regulators of Platelet Activation. Int. J. Mol. Sci..

[54] Mandel J., Casari M., Stepanyan M., Martyanov A., Deppermann C. (2022). Beyond Hemostasis: Platelet Innate Immune Interactions and Thromboinflammation. Int. J. Mol. Sci..

[55] Koupenova M., Livada A.C., Morrell C.N. (2022). Platelet and Megakaryocyte Roles in Innate and Adaptive Immunity. Circ. Res..

[56] Maouia A., Rebetz J., Kapur R., Semple J.W. (2020). The Immune Nature of Platelets Revisited. Transfus. Med. Rev..

[57] Tsygankov A.Y. (2013). TULA-family proteins: A new class of cellular regulators. J. Cell Physiol..

[58] Tsygankov A.Y. (2019). TULA-family proteins: Jacks of many trades and then some. J. Cell Physiol..

[59] Luis B.S., Carpino N. (2014). Insights into the suppressor of T-cell receptor (TCR) signaling-1 (Sts-1)-mediated regulation of TCR signaling through the use of novel substrate-trapping Sts-1 phosphatase variants. FEBS J..

[60] Lupher M.L., Rao N., Lill N.L., Andoniou C.E., Miyake S., Clark E.A., Druker B., Band H. (1998). Cbl-mediated negative regulation of the Syk tyrosine kinase. A critical role for Cbl phosphotyrosine-binding domain binding to Syk phosphotyrosine 323. J. Biol. Chem..

[61] Ota S., Hazeki K., Rao N., Lupher M.L., Andoniou C.E., Druker B., Band H. (2000). The RING finger domain of Cbl is essential for negative regulation of the Syk tyrosine kinase. J. Biol. Chem..

[62] Rao N., Ghosh A.K., Ota S., Zhou P., Reddi A.L., Hakezi K., Druker B.K., Wu J., Band H. (2001). The non-receptor tyrosine kinase Syk is a target of Cbl-mediated ubiquitylation upon B-cell receptor stimulation. EMBO J..

[63] Goyama S., Schibler J., Gasilina A., Shrestha M., Lin S., Link K.A., Chen J., Whitman S.P., Bloomfield C.D., Nicolet D. (2016). UBASH3B/Sts-1-CBL axis regulates myeloid proliferation in human preleukemia induced by AML1-ETO. Leukemia.

[64] Lee S.T., Feng M., Wei Y., Li Z., Qiao Y., Guan P., Jiang X., Wong C.H., Huynh K., Wang J. (2013). Protein tyrosine phosphatase UBASH3B is overexpressed in triple-negative breast cancer and promotes invasion and metastasis. Proc. Natl. Acad. Sci. USA.

[65] Raguz J., Wagner S., Dikic I., Hoeller D. (2007). Suppressor of T-cell receptor signalling 1 and 2 differentially regulate endocytosis and signalling of receptor tyrosine kinases. FEBS Lett..

[66] Zhang J., Vakhrusheva O., Bandi S.R., Demirel O., Kazi J.U., Fernandes R.G., Jakobi K., Eichler A., Ronnstrand L., Rieger M.A. (2015). The phosphatases STS1 and STS2 regulate hematopoietic stem and progenitor cell fitness. Stem Cell Rep..

[67] Yan Q., Barros T., Visperas P.R., Deindl S., Kadlecek T.A., Weiss A., Kuriyan J. (2013). Structural basis for activation of ZAP-70 by phosphorylation of the SH2-kinase linker. Mol. Cell. Biol..

[68] Hong J.J., Yankee T.M., Harrison M.L., Geahlen R.L. (2002). Regulation of signaling in B cells through the phosphorylation of Syk on linker region tyrosines. A mechanism for negative signaling by the Lyn tyrosine kinase. J. Biol. Chem..

[69] Simon M., Vanes L., Geahlen R.L., Tybulewicz V.L. (2005). Distinct roles for the linker region tyrosines of Syk in FcepsilonRI signaling in primary mast cells. J. Biol. Chem..

[70] Groesch T.D., Zhou F., Mattila S., Geahlen R.L., Post C.B. (2006). Structural basis for the requirement of two phosphotyrosine residues in signaling mediated by Syk tyrosine kinase. J. Mol. Biol..

[71] Tsang E., Giannetti A.M., Shaw D., Dinh M., Tse J.K., Gandhi S., Ho H., Wang S., Papp E., Bradshaw J.M. (2008). Molecular mechanism of the Syk activation switch. J. Biol. Chem..

[72] Gradler U., Schwarz D., Dresing V., Musil D., Bomke J., Frech M., Greiner H., Jakel S., Rysiok T., Muller-Pompalla D. (2013). Structural and biophysical characterization of the Syk activation switch. J. Mol. Biol..

[73] Zhang J., Berenstein E., Siraganian R.P. (2002). Phosphorylation of Tyr342 in the linker region of Syk is critical for Fc epsilon RI signaling in mast cells. Mol. Cell. Biol..

[74] Chen C.H., Martin V.A., Gorenstein N.M., Geahlen R.L., Post C.B. (2011). Two closely spaced tyrosines regulate NFAT signaling in B cells via Syk association with Vav. Mol. Cell. Biol..

[75] Wu J., Zhao Q., Kurosaki T., Weiss A. (1997). The Vav binding site (Y315) in ZAP-70 is critical for antigen receptor-mediated signal transduction. J. Exp. Med..

[76] Di Bartolo V., Mege D., Germain V., Pelosi M., Dufour E., Michel F., Magistrelli G., Isacchi A., Acuto O. (1999). Tyrosine 319, a newly identified phosphorylation site of ZAP-70, plays a critical role in T cell antigen receptor signaling. J. Biol. Chem..

[77] Kostyak J.C., Mauri B., Dangelmaier C., Vari H.R., Patel A., Wright M., Reddy H., Tsygankov A.Y., Kunapuli S.P. (2022). Phosphorylation on Syk Y342 is important for both ITAM and hemITAM signaling in platelets. J. Biol. Chem..

[78] Mansueto M.S., Reens A., Rakhilina L., Chi A., Pan B.S., Miller J.R. (2019). A reevaluation of the spleen tyrosine kinase (SYK) activation mechanism. J. Biol. Chem..

[79] Dangelmaier C.A., Quinter P.G., Jin J., Tsygankov A.Y., Kunapuli S.P., Daniel J.L. (2005). Rapid ubiquitination of Syk following GPVI activation in platelets. Blood.

[80] Lupher M.L., Songyang Z., Shoelson S.E., Cantley L.C., Band H. (1997). The Cbl phosphotyrosine-binding domain selects a D(N/D)XpY motif and binds to the Tyr292 negative regulatory phosphorylation site of ZAP-70. J. Biol. Chem..

[81] Rao N., Lupher M.L., Ota S., Reedquist K.A., Druker B.J., Band H. (2000). The linker phosphorylation site Tyr292 mediates the negative regulatory effect of Cbl on ZAP-70 in T cells. J. Immunol..

[82] Teckchandani A.M., Birukova A.A., Tar K., Verin A.D., Tsygankov A.Y. (2005). The multidomain protooncogenic protein c-Cbl binds to tubulin and stabilizes microtubules. Exp. Cell Res..

[83] Lozic M., Minarik L., Racetin A., Filipovic N., Saraga Babic M., Vukojevic K. (2021). CRKL, AIFM3, AIF, BCL2, and UBASH3A during Human Kidney Development. Int. J. Mol. Sci..

[84] Smidak R., Mayer R.L., Bileck A., Gerner C., Mechtcheriakova D., Stork O., Lubec G., Li L. (2016). Quantitative proteomics reveals protein kinases and phosphatases in the individual phases of contextual fear conditioning in the C57BL/6J mouse. Behav. Brain Res..

[85] Grant S.F., Qu H.Q., Bradfield J.P., Marchand L., Kim C.E., Glessner J.T., Grabs R., Taback S.P., Frackelton E.C., Eckert A.W. (2009). Follow-up analysis of genome-wide association data identifies novel loci for type 1 diabetes. Diabetes.

[86] Plagnol V., Howson J.M., Smyth D.J., Walker N., Hafler J.P., Wallace C., Stevens H., Jackson L., Simmonds M.J., Bingley P.J. (2011). Genome-wide association analysis of autoantibody positivity in type 1 diabetes cases. PLoS Genet..

[87] Zhernakova A., Stahl E.A., Trynka G., Raychaudhuri S., Festen E.A., Franke L., Westra H.J., Fehrmann R.S., Kurreeman F.A., Thomson B. (2011). Meta-analysis of genome-wide association studies in celiac disease and rheumatoid arthritis identifies fourteen non-HLA shared loci. PLoS Genet.

[88] Diaz-Gallo L.M., Sanchez E., Ortego-Centeno N., Sabio J.M., Garcia-Hernandez F.J., de Ramon E., Gonzalez-Gay M.A., Torsten W., Anders H.J., Gonzalez-Escribano M.F. (2013). Evidence of new risk genetic factor to systemic lupus erythematosus: The UBASH3A gene. PLoS ONE.

[89] Kim K., Bang S.Y., Lee H.S., Cho S.K., Choi C.B., Sung Y.K., Kim T.H., Jun J.B., Yoo D.H., Kang Y.M. (2015). High-density genotyping of immune loci in Koreans and Europeans identifies eight new rheumatoid arthritis risk loci. Ann. Rheum. Dis..

[90] Liu J., Liu J., Ni J., Leng R.X., Pan H.F., Ye D.Q. (2015). Association of UBASH3A gene polymorphisms and systemic lupus erythematosus in a Chinese population. Gene.

[91] Ji S.G., Juran B.D., Mucha S., Folseraas T., Jostins L., Melum E., Kumasaka N., Atkinson E.J., Schlicht E.M., Liu J.Z. (2017). Genome-wide association study of primary sclerosing cholangitis identifies new risk loci and quantifies the genetic relationship with inflammatory bowel disease. Nat. Genet..

[92] Li Y., Cheng H., Xiao F.L., Liang B., Zhou F.S., Li P., Zheng X.D., Sun L.D., Yang S., Zhang X.J. (2017). Association of UBASH3A gene polymorphism and atopic dermatitis in the Chinese Han population. Genes Immun..

[93] Liu J., Ni J., Li L.J., Leng R.X., Pan H.F., Ye D.Q. (2015). Decreased UBASH3A mRNA Expression Levels in Peripheral Blood Mononuclear Cells from Patients with Systemic Lupus Erythematosus. Inflammation.

[94] Steck A.K., Wong R., Wagner B., Johnson K., Liu E., Romanos J., Wijmenga C., Norris J.M., Eisenbarth G.S., Rewers M.J. (2012). Effects of non-HLA gene polymorphisms on development of islet autoimmunity and type 1 diabetes in a population with high-risk HLA-DR,DQ genotypes. Diabetes.

[95] Johnson K., Wong R., Barriga K.J., Klingensmith G., Ziegler A.G., Rewers M.J., Steck A.K. (2012). rs11203203 is associated with type 1 diabetes risk in population pre-screened for high-risk HLA-DR,DQ genotypes. Pediatr. Diabetes.

[96] Concannon P., Onengut-Gumuscu S., Todd J.A., Smyth D.J., Pociot F., Bergholdt R., Akolkar B., Erlich H.A., Hilner J.E., Julier C. (2008). A human type 1 diabetes susceptibility locus maps to chromosome 21q22.3. Diabetes.

[97] Fei Y., Webb R., Cobb B.L., Direskeneli H., Saruhan-Direskeneli G., Sawalha A.H. (2009). Identification of novel genetic susceptibility loci for Behcet’s disease using a genome-wide association study. Arthritis Res. Ther..

[98] Shahriyari E., Bonyadi M., Jabbarpoor Bonyadi M.H., Soheilian M., Yaseri M., Ebrahimiadib N. (2019). Ubiquitin Associated and SH3 Domain Containing B (UBASH3B) Gene Association with Behcet’s Disease in Iranian Population. Curr. Eye Res..

[99] Ge Y., Concannon P. (2018). Molecular-genetic characterization of common, noncoding UBASH3A variants associated with type 1 diabetes. Eur. J. Hum. Genet..

[100] Parashar K., Kopping E., Frank D., Sampath V., Thanassi D.G., Carpino N. (2017). Increased Resistance to Intradermal Francisella tularensis LVS Infection by Inactivation of the Sts Phosphatases. Infect. Immun..

[101] Zhou W., Yin Y., Smith E., Chou J., Shumate J., Scampavia L., Spicer T.P., Carpino N., French J.B. (2019). Discovery and Characterization of Two Classes of Selective Inhibitors of the Suppressor of the TCR Signaling Family of Proteins. ACS Infect. Dis..

[102] Li N., Wang Y., Wang A., Zhang J., Jia C., Yu C., Song Z., Wang S., Liu L., Yi J. (2023). STS1 and STS2 Phosphatase Inhibitor Baicalein Enhances the Expansion of Hematopoietic and Progenitor Stem Cells and Alleviates 5-Fluorouracil-Induced Myelosuppression. Int. J. Mol. Sci..

[103] Papadopoulos J.S., Agarwala R. (2007). COBALT: Constraint-based alignment tool for multiple protein sequences. Bioinformatics.

[104] Grishin N.V. (1995). Estimation of the number of amino acid substitutions per site when the substitution rate varies among sites. J. Mol. Evol..

[105] Kasahara M. (2007). The 2R hypothesis: An update. Curr. Opin. Immunol..

[106] Chen Y., Jakoncic J., Wang J., Zheng X., Carpino N., Nassar N. (2008). Structural and functional characterization of the c-terminal domain of the ecdysteroid phosphate phosphatase from bombyx mori reveals a new enzymatic activity. Biochemistry.

[107] Davies L., Anderson I.P., Turner P.C., Shirras A.D., Rees H.H., Rigden D.J. (2007). An unsuspected ecdysteroid/steroid phosphatase activity in the key T-cell regulator, Sts-1: Surprising relationship to insect ecdysteroid phosphate phosphatase. Proteins.

[108] Yamada R., Sonobe H. (2003). Purification, kinetic characterization, and molecular cloning of a novel enzyme ecdysteroid-phosphate phosphatase. J. Biol. Chem..

[109] Asada M., Kato Y., Matsuura T., Watanabe H. (2014). Early embryonic expression of a putative ecdysteroid-phosphate phosphatase in the water flea, Daphnia magna (Cladocera: Daphniidae). J. Insect. Sci..

[110] Rosental B., Raveh T., Voskoboynik A., Weissman I.L. (2020). Evolutionary perspective on the hematopoietic system through a colonial chordate: Allogeneic immunity and hematopoiesis. Curr. Opin. Immunol..

[111] Yuan S., Tao X., Huang S., Chen S., Xu A. (2014). Comparative immune systems in animals. Annu. Rev. Anim. Biosci..

[112] Soderhall K. (2010). Invertebrate Immunity.

[113] Morales Poole J.R., Paganini J., Pontarotti P. (2017). Convergent evolution of the adaptive immune response in jawed vertebrates and cyclostomes: An evolutionary biology approach based study. Dev. Comp. Immunol..

[114] Reiber C., McGaw I. (2009). A review of the “open” and “closed” circulatory systems: New terminology for complex invertebrate circulatory systems in light of current findings. Int. J. Zool..

[115] Piavis G., Hiatt J.L. (1971). Blood cell lineage in the sea lamprey, *Petromyzon marinus* (Pisces: Petromyzontidae). Copeia.

[116] Jordan H., Speidel C. (1930). Blood formation in cyclostomes. Am. J. Anat..

[117] Weyand A.C., Shavit J.A. (2014). Zebrafish as a model system for the study of hemostasis and thrombosis. Curr. Opin. Hematol..

[118] Hyder S.L., Cayer M.L., Pettey C.L. (1983). Cell types in peripheral blood of the nurse shark: An approach to structure and function. Tissue Cell.

[119] Lee K.G., Miller T., Anastassov I., Cohen W.D. (2004). Shape transformation and cytoskeletal reorganization in activated non-mammalian thrombocytes. Cell Biol. Int..

[120] Pica A., Lodato A., Grimaldi M.C., Della Corte F. (1990). Morphology, origin and functions of the thrombocytes of Elasmobranchs. Arch. Ital. Anat. Embriol..

[121] Jagadeeswaran P., Sheehan J.P., Craig F.E., Troyer D. (1999). Identification and characterization of zebrafish thrombocytes. Br. J. Haematol..

[122] Hill D.J., Griffiths D.H., Rowley A.F. (1999). Trout thrombocytes contain 12- but not 5-lipoxygenase activity. Biochim. Biophys. Acta.

[123] Buonocore F., Gerdol M. (2016). Alternative adaptive immunity strategies: Coelacanth, cod and shark immunity. Mol. Immunol..

[124] Flajnik M.F. (2014). Re-evaluation of the immunological Big Bang. Curr. Biol..

[125] Flajnik M.F. (2018). A cold-blooded view of adaptive immunity. Nat. Rev. Immunol..

[126] Das S., Li J., Hirano M., Sutoh Y., Herrin B.R., Cooper M.D. (2015). Evolution of two prototypic T cell lineages. Cell Immunol..

[127] Boehm T., McCurley N., Sutoh Y., Schorpp M., Kasahara M., Cooper M.D. (2012). VLR-based adaptive immunity. Annu. Rev. Immunol..

[128] Boehm T., Hirano M., Holland S.J., Das S., Schorpp M., Cooper M.D. (2018). Evolution of Alternative Adaptive Immune Systems in Vertebrates. Annu. Rev. Immunol..

[129] Mitchell C.D., Criscitiello M.F. (2020). Comparative study of cartilaginous fish divulges insights into the early evolution of primary, secondary and mucosal lymphoid tissue architecture. Fish Shellfish Immunol..

[130] Mocsai A., Ruland J., Tybulewicz V.L. (2010). The SYK tyrosine kinase: A crucial player in diverse biological functions. Nat. Rev. Immunol..

[131] Geahlen R.L. (2009). Syk and pTyr’d: Signaling through the B cell antigen receptor. Biochim. Biophys. Acta.

[132] Au-Yeung B.B., Shah N.H., Shen L., Weiss A. (2017). ZAP-70 in Signaling, Biology, and Disease. Annu. Rev. Immunol..

[133] Latour S., Chow L.M., Veillette A. (1996). Differential intrinsic enzymatic activity of Syk and Zap-70 protein-tyrosine kinases. J. Biol. Chem..

